# Curved Bistable Origami‐Inspired Flexible Transcatheter Mitral Valve Clamping

**DOI:** 10.1002/advs.202517350

**Published:** 2025-11-25

**Authors:** Siyu Gao, Fan Jiang, Xiuting Sun, Peng Qi, Jian Xu

**Affiliations:** ^1^ School of Aerospace Engineering and Applied Mechanics Tongji University Shanghai 201800 China; ^2^ Mode Key Laboratory of Autonomous Intelligent Unmanned Systems Shanghai 201800 China; ^3^ Department of Control Science and Engineering College of Electronics and Information Engineering Tongji University Shanghai 200082 China

**Keywords:** bistable curved origami, curvature of space, interventional medical device, stiffness design, transcatheter mitral valve clamping

## Abstract

Curved origami exhibits remarkable potential for minimally invasive medical applications owing to its unique geometric programmability and mechanical tunability. Building on this concept, a curved origami with bistable mechanical behavior is proposed, specifically engineered for transcatheter mitral valve clamping surgery. Benefiting from its bistable characteristics, the curved origami structure enables three key functions: it remains compactly folded during catheter delivery, self‐deploys rapidly and precisely at the target site, and maintains a stable, fully expanded configuration during device retrieval. The equivalent stiffness of the curved origami mechanism is tuned through geometric nonlinearity, ensuring that its critical transition load closely simulates the catheter‐based delivery conditions. Theoretical analysis and experimental validation confirm that bistability is effectively preserved, while parameters such as initial curvature, thickness, crease curvature, and segment length can be adjusted to precisely tailor the radial support stiffness, meeting the mechanical requirements for resisting mitral valve contraction forces. In vitro experiments further verify the dilator's exceptional stability and fully reliable deployment within a simulated mitral valve environment. This work advances the engineering application of curved origami mechanics in minimally invasive medical treatments, offering both theoretical insights and promising potential for clinical translation.

## Graphical abstract

A lightweight bistable curved origami dilator combines reversible deformation and high stability, offering a promising solution for minimally invasive mitral valve repair.

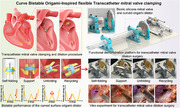

## 1 Introduction

Mitral regurgitation (MR) is one of the most common cardiovascular diseases.^[^ ^1^ ^]^ Treatment has progressively shifted from open‐heart surgery to minimally invasive transcatheter mitral valve repair (TMVR), which markedly improves the prognosis of high‐risk patients owing to its reduced invasiveness.^[^ ^2^, ^3^, ^4^, ^5^ ^]^ However, transcatheter mitral valve clamping (TMVC) devices still face challenges such as limitations in delivery size and difficulties in achieving precise deployment and reliable positioning of the gripper within the complex mitral valve environment.^[^ ^6^, ^7^ ^]^ Origami engineering offers innovative solutions to these challenges by leveraging its geometric reconfigurability and foldable deformation characteristics^[^ ^8^, ^9^ ^]^ to inspire novel designs for deployable TMVC devices. By tuning the geometric parameters of the origami, controlled bistable mechanical behavior can be achieved—allowing the device to remain in a compact, semi‐stable configuration during catheter delivery and then reliably unfold into a stable, functional configuration at the target site. This bistability provides sufficient radial support to withstand the dynamic loading conditions of the beating heart.

Origami engineering originated from the scientific reconstruction of geometric transformation mechanisms in the traditional art of origami.^[^ ^10^ ^]^ Since the mathematical modeling of fundamental configurations such as the Miura‐ori, cross‐scale engineering applications have emerged through advancements in topological parameterization methods. These developments have led to deployable aerospace structures,^[^ ^11^, ^12^ ^]^ self‐folding robots,^[^ ^13^, ^14^, ^15^, ^16^ ^]^ biomedical devices,^[^ ^17^, ^18^ ^]^ micro‐ and nano‐folded components,^[^ ^19^, ^20^, ^21^ ^]^ and adaptive sensors,^[^ ^22^ ^]^ with origami engineering evolving into a comprehensive, multilevel technological system. Origami engineering employs geometric and topological design principles to create diverse mechanical regulation mechanisms within bistable or multistable structures^[^ ^23^, ^24^ ^]^ and metamaterials.^[^ ^25^, ^26^, ^27^ ^]^ It is defined by the presence of two or more stable equilibrium modes: when subjected to an external force, they can undergo reversible transitions between distinct configurations, and once the force is removed, they remain in the new stable mode. This regulatory mechanism endows multistable structures with excellent environmental adaptability and enables them to autonomously maintain their configurations under varying operating conditions, without continuous energy input. Notably, origami structures often exhibit different mechanical dynamics in each of their stable modes. This property enables the integration of multiple functionalities in a wide range of engineering applications, such as sensing, vibration isolation, and energy harvesting.^[^ ^28^, ^29^, ^30^, ^31^, ^32^ ^]^

In recent years, significant progress has been made in the study of bistable and multi‐stable properties of origami engineering.^[^ ^33^ ^]^ For example, the stacking of multiple Miura‐ori cells can achieve a static mechanical diode effect through asymmetric energy barriers.^[^ ^34^ ^]^ The Kresling cylindrical origami structure exhibits unique compression–torsion coupling bistability, arising from the combined effects of bending deformation in the folded surfaces and torsional deformation along the creases.^[^ ^35^ ^]^ Origami spheres constructed from 6‐vertex Waterbomb cells demonstrate axial multistability and radial flexibility.^[^ ^36^ ^]^ Interestingly, traditional origami patterns such as the square twist—although possessing zero degrees of freedom (DOF) in their crease patterns and therefore being theoretically rigid and non‐foldable—can still undergo folding by exploiting out‐of‐plane bending deformations that are not captured by rigid‐crease models.^[^ ^37^ ^]^ As a result, most origami configurations are non‐foldable under rigid‐folding conditions, which limits their applicability in scenarios requiring high flexibility and large deformation. Curved origami addresses this limitation by coupling surface elasticity with crease rotation, thereby achieving higher deformability and additional degrees of freedom for stable‐mode regulation.^[^ ^38^, ^39^, ^40^ ^]^

With continued research, curved origami has demonstrated remarkable properties, including tunable Poisson's ratio, programmable stiffness, and impact absorption capacity. For example, mechanical metamaterials based on curved crease origami (CCO) exhibit both tunable Poisson's ratio and stiffness,^[^ ^41^ ^]^ meanwhile, curved origami‐inspired materials with elastic curvature influence nonlinear force–displacement behavior.^[^ ^42^ ^]^ Additionally, composite sandwich structures incorporating curved‐crease origami cores exhibit superior performance under low‐velocity impact conditions.^[^ ^43^ ^]^ When combined with cellular lattices, origami reveals unique compressive behavior of bent creases and prismatic structures with varying cross‐sections.^[^ ^44^ ^]^ Bent origami metamaterials with gradient and composite reinforcement provide excellent energy absorption and load‐bearing capacity.^[^ ^45^ ^]^ Under uniform load, bent origami enables versatile gripping, controlled force transfer, and multilevel stiffness.^[^ ^46^ ^]^ Owing to its enhanced design flexibility, curved origami exhibits a broader range of tunable properties and expands its potential applications to adaptive medical devices, flexible robotics, and smart metamaterials.^[^ ^47^, ^48^, ^49^ ^]^

**Figure 1:**
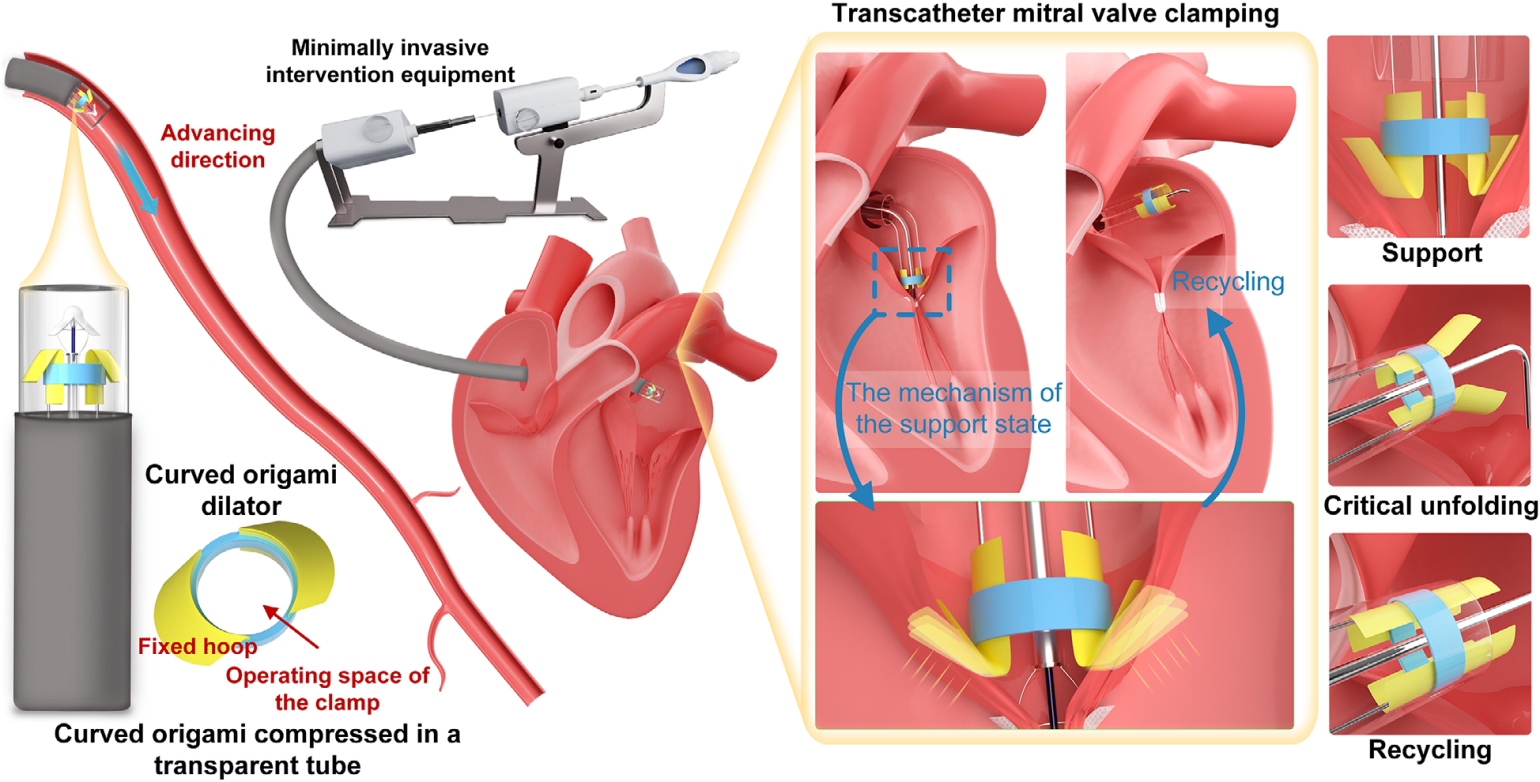
Working mechanism of the bistable curved origami dilator of TMVC:Model of transcatheter mitral valve curved origami dilator and its direction of propulsion; Detail of the curved origami dilator; Schematic diagram of transcatheter mitral valve clamping surgery; Three modes of bistable curved origami.

Based on the prior research, we propose a curved origami dilator for TMVC, as shown in **Figure** **1**. In its self‐folding mode (the left side of Figure 1), it remains compact for catheter delivery. Upon release, it unfolds to provide high radial stiffness that resists valve contraction, then passes through an unstable transition point during retrieval before reaching a fully unfolded mode for safe extraction. The right side of Figure 1 outlines the TMVC surgery, where repeated valve motion complicates clamp placement. The curved origami dilator addresses this by stabilizing the valve, allowing smooth clamp passage through the fixed hoop, and is withdrawn once the valve is secured.

To facilitate the development of adaptable mitral valve dilators, we established a mechanical model of curved origami and analyzed the key parameters affecting its bistability and radial stiffness. Using a biomimetic mitral valve composed of multi‐stiffness silicone, we evaluated the support stability and release reliability of the curved origami dilator on a simulated TMVC platform. The full delivery, unfolding, and retrieval process was successfully simulated in an ex vivo porcine heart, exhibiting excellent morphological stability and a 100% deployment success rate. This work provides a practical foundation for the design of deployable curved origami structures in interventional medical devices.

## 2 Results and Discussion

### 2.1 Mechanical Model of Curved Origami Dilator

**Figure 2:**
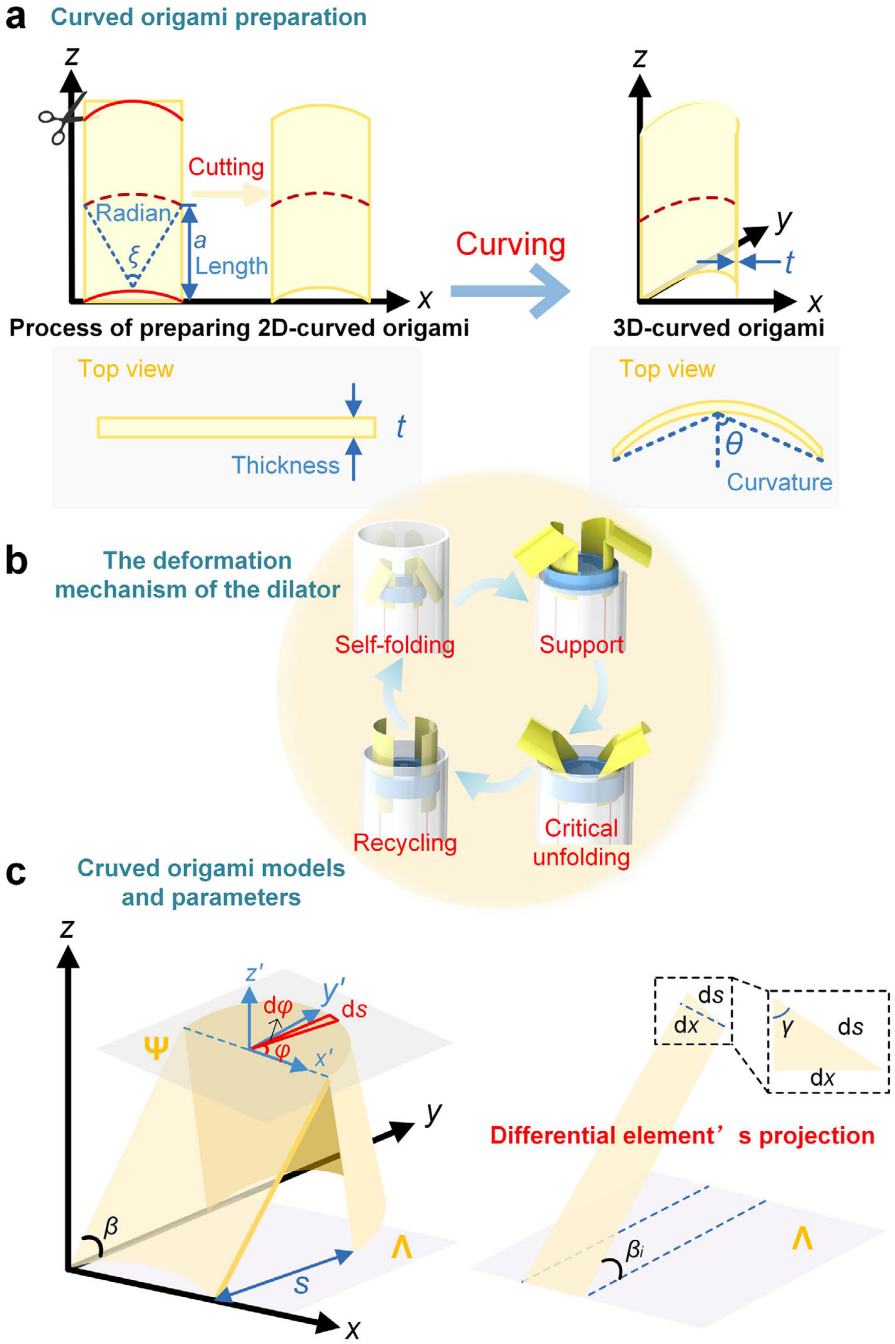
Mechanical model of curved origami dilator: a) The preparation process of curved origami; b) Curved origami folding mechanism; c) Curved origami adjustable parameters and schematic of differential element's projection.

**Figure** **2** illustrates the design strategy, functional mechanism, and geometric modeling of the curved origami dilator. As shown in Figure 2a, a 2D curved origami unit is obtained by cutting a flat sheet of paper with a constant thickness of *t*. Its initial geometric shape is composed of a circular arc with a central angle of ξ and a rectangle with length *a*. Then, this 2D structure is transformed into a 3D curved origami by bending along the *y*‐axis in the plane, thereby introducing the spatial curvature θ. The top view further illustrates the evolution of the curved origami from a straight line to a curved shape, and the design of the curved origami dilator is achieved through the initial geometric design and bending.

Figure 2b illustrates the deformation cycle of the curved origami dilator, comprising four key functional morphologies: self‐folding, support, critical unfolding, and recycling. Initially, the dilator undergoes self‐folding, enabling its actuation along the tube. Upon exiting the constrained environment of the tube, the dilator enters the support phase, during which it comes into contact with the mitral valve and provides mechanical stability. When a force opposite to the pushing direction is applied, the dilator comes into contact with the tube wall and reaches an unstable critical unfolding mode. Once this critical point is crossed, the dilator will fully unfold and be able to recover in accordance with the direction of the tube wall. To establish a theoretical foundation for stiffness design and adjustment during the operation of the curved origami dilator, we propose a mechanical modeling approach to elucidate its deformation mechanisms and bistable behavior.

Figure 2c illustrates the general model of the curved origami dilator. During deformation, the surfaces between adjacent creases are assumed to undergo pure bending. Using a differential approach, the curved origami is discretized into *N* units, each with an arc length d*s* and width d*x*. In the 3D schematic in Figure 2c, a local coordinate system (*x*′,  *y*′,  *z*′) is established with the origin at arc center. The vector from the arc's starting point forms an angle φ with the *x*′‐axis, and the vector at the arc's endpoint forms an angle φ  +  dφ. The angle γ between the differential unit arc and the edge line is expressed as:

(1) $$\gamma =\arcsin\sqrt{\sin^{2}\beta +\cos^{2}\beta \cos^{2}\varphi }$$

where arc length d*s* is expressed as:

(2) $$ds=\frac{a}{\cos\theta }d\varphi$$

The folding‐induced deformation of curved origami is characterized by the angle β between the edge line and the *xy*‐plane. Based on this assumption, the dimensional variation of the curved origami in the radial direction can be expressed as:

(3) s=2asinβ

The overall deformation behavior of the curved origami is modeled based on the principle of minimum potential energy. The total potential energy of the structure can be expressed as:

(4) π=U−V

In Equation (4), *V* represents the work done by the external force as:

(5) $$V=\int_{s_{0}}^{s}f_{s}ds$$

and *U* denotes the total potential energy, comprising both the bending energy along the crease lines and the in‐plane strain energy of the origami, as:

(6) $$U=\frac{1}{2}\int_{0}^{s_{0}}k{\left(\alpha -\alpha_{0}\right)}^{2}ds+2\int_{0}^{s_{0}}\frac{EI}{2}{\left(\kappa -\kappa_{0}\right)}^{2}ds$$

In the potential energy function, *k* denotes the rotational stiffness of the crease, *E* is the elastic modulus of the curved origami material, *I* represents the cross‐sectional moment of inertia within the origami surface plane, and *EI* corresponds to the in‐plane bending stiffness, α is the dihedral angle along the crease, while κ indicates the curvature in the out‐of‐plane bending direction.

In an arbitrary differential unit, the dihedral angle α_*i*_ (where *i* represents the *i*‐th differential unit) of the crease of the folded curve can be expressed as:

(7) $$\alpha_{i}=\pi -2\beta_{i}$$

where β_*i*_ represents the dihedral angle between the differential unit and the *x‐y* plane. Therefore, the dihedral angle β between the curve folding and the *x‐y* plane can be obtained as:

(8) $$\sin\beta =\sin\beta_{i}\sin\gamma$$

Combining Equations (7) and (8), the dihedral angle of crease is obtained as:

(9) $$\alpha =\pi -2\arcsin\left(\frac{\sin\beta }{\sin\gamma }\right)$$

According to the 3D graph analysis in Figure 2c, assuming that the arc of the curvature surface is expressed by the function *f*  =  *g*(*x*), the relationship between the curvature κ after the curve is folded and the slope *g*′(*x*) of the arc can be expressed as:

(10) $$\kappa =\frac{g^{\prime \prime }\left(x\right)}{{\left[1+g^{\prime }{\left(x\right)}^{2}\right]}^{\frac{3}{2}}}$$

and the relationship between *g*′(*x*) and the central angle φ is expressed as:

(11) $$g^{\prime }\left(x\right)=2\sin\gamma \tan\varphi d\varphi$$

Substituting Equations (9) and (11) into Equation (6) yields the potential energy function for the curve folding with all parameters during deformation.

### 2.2 Bistable Performance and Designable Stiffness

**Figure 3:**
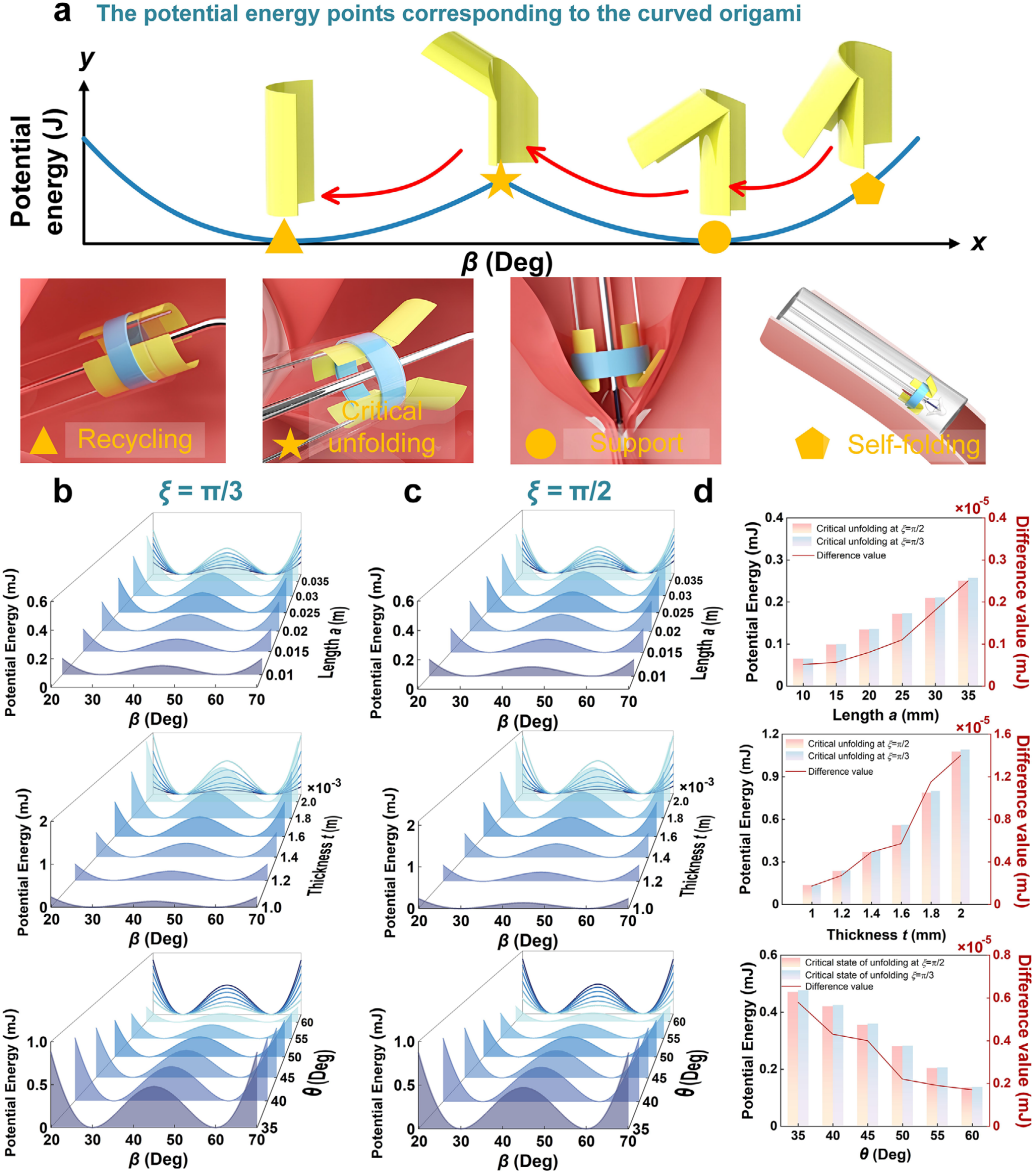
Bistable folding mechanism and energy analysis of curved origami dilator: a) Folding–unfolding transition mechanism illustrating four key modes: self‐folding, support, critical unfolding and recycling; b, c) Potential energy of curved origami dilator under varying geometric parameters with crease radian of ξ  =  π/3 and ξ  =  π/2, respectively; (d) Quantitative comparison of potential energy of curved origami differences at critical unfolding mode with crease radian of ξ  =  π/3 and ξ  =  π/2, respectively.

To adaptively apply bistable behavior during deployment, valve interaction, and recycling, the curved origami dilator must exhibit different stiffness under varying out‐of‐plane loads. Therefore, we established a folding–unfolding transition model, as illustrated in **Figure** **3**a. When the curved origami dilator moves within the tube under the constraint of the tube wall, it remains in a self‐folding mode, which serves as a potential energy storage mode, corresponding to a self‐folding configuration. Once the dilator is pushed out of the tube, it immediately transitions into the supporting mode, where it comes into contact with the mitral valve. Through its radial stiffness, the dilator resists the retraction pressure of the valve. Through its radial stiffness, the dilator resists the retraction pressure of the mitral valve. This supports the valve leaflets, facilitates clamp operation, minimizes clamp‐valve tissue interference, and enhances surgical stability and safety. During valve clamping, the dilator is retracted and undergoes a critical unfolding transition (critical mode), which is driven by the interaction force exerted by the tube opening on its curved origami. Ultimately, the dilator fully returns into the tube, which reaches recycling mode, completing a full deformation‐recovery cycle. The pathway marked with arrows and symbols on the panel of Figure 3a reflects the actual actuation trajectory of the dilator through different energy modes. Among them, the pentagon, circle, pentagram, and triangle markers represent the self‐folding mode, support mode, critical unfolding mode, and recycling mode. This process demonstrates excellent mechanical reversibility and structural stability, forming a fully self‐adaptive and closed‐loop transition pathway.

Figure 3b,c shows how different geometric parameters affect the potential deformation energy of the curved origami dilator with crease radian ξ  =  π/3 and ξ  =  π/2, respectively. Figure 3b,c depict potential energy curves for variations in length, thickness, and curvature, respectively. As shown in the results, increasing the length deepens the potential energy well and raises the energy barrier between the two stable modes, thereby enhancing the bistable effect. Similarly, increasing thickness also significantly raises the potential energy barrier. In contrast, increasing the curvature angle shifts the stable mode positions and lowers the energy barrier, indicating that curvature plays a crucial role in tuning the bistable behavior of curved origami.

Furthermore, Figure 3d compares the variations of critical potential deformation energy for the curved origami dilator at crease radians ξ  =  π/3 and ξ  =  π/2. It presents histograms of critical potential energy for variations in length, thickness, and curvature, respectively. The results indicate that increasing either the length or the thickness causes a significant rise in the energy barrier between the two stable modes of the potential deformation energy curve, thereby improving both mode stability and overall structural robustness. While the crease radius has a relatively smaller influence on bistability, a slight decrease in the potential energy barrier is still observed as the radius increases. Curved origami dilator benefits from being lightweight, high compression ratio, reversible folding‐unfolding behavior, and structural stability.

In order to ensure that the dilator can provide sufficient radial support stiffness during the interaction process with the mitral valve, and the tube wall can provide sufficient restoring force for the dilator during the recycle process, we designed the variation of stiffness of the curved origami dilator by using the potential energy method. The equivalent stiffness of the curved origami dilator is derived to characterize the restoring force response during deformation. By applying the equilibrium condition $\frac{d\pi }{d\beta }=0$ to total potential deformation energy expression as Equation (4), the following relationship is obtained:

(12) $$f_{s}\frac{ds}{d\beta }=\frac{dU}{d\beta }$$

In order to support the heart valve, it requires sufficient support stiffness at the targeted position. The loading on the annular surface applied parallel to the radial direction is considered. The radial force of the curved origami dilator during the whole deformation process can be written as

(13) $$f_{s}=\frac{dU/d\beta }{ds/d\beta }$$

As the same consideration of the definition of equivalent support force as Equation (13), the equivalent stiffness is determined by the derivative of the genialized with respect to the genialized displacement *β*, i.e., the second derivative of the potential energy function, defined as:

(14) $$K_{s}=\frac{df_{s}/d\beta }{ds/d\beta }$$

The stability of the system can be judged according to the sign of the equivalent stiffness. When the derivative in Equation (14) is positive, i.e.:

(15) $$\partial^{2}U/d\beta^{2}>0$$

The system is in a stable equilibrium state where small perturbations are resisted by a restoring moment. When $\frac{d^{2}U}{d\beta^{2}}=0$, the system reaches a critical point that marks the onset of instability. In contrast, a negative value $\frac{d^{2}U}{d\beta^{2}}<0$ corresponds to a locally unstable region.

**Figure 4:**
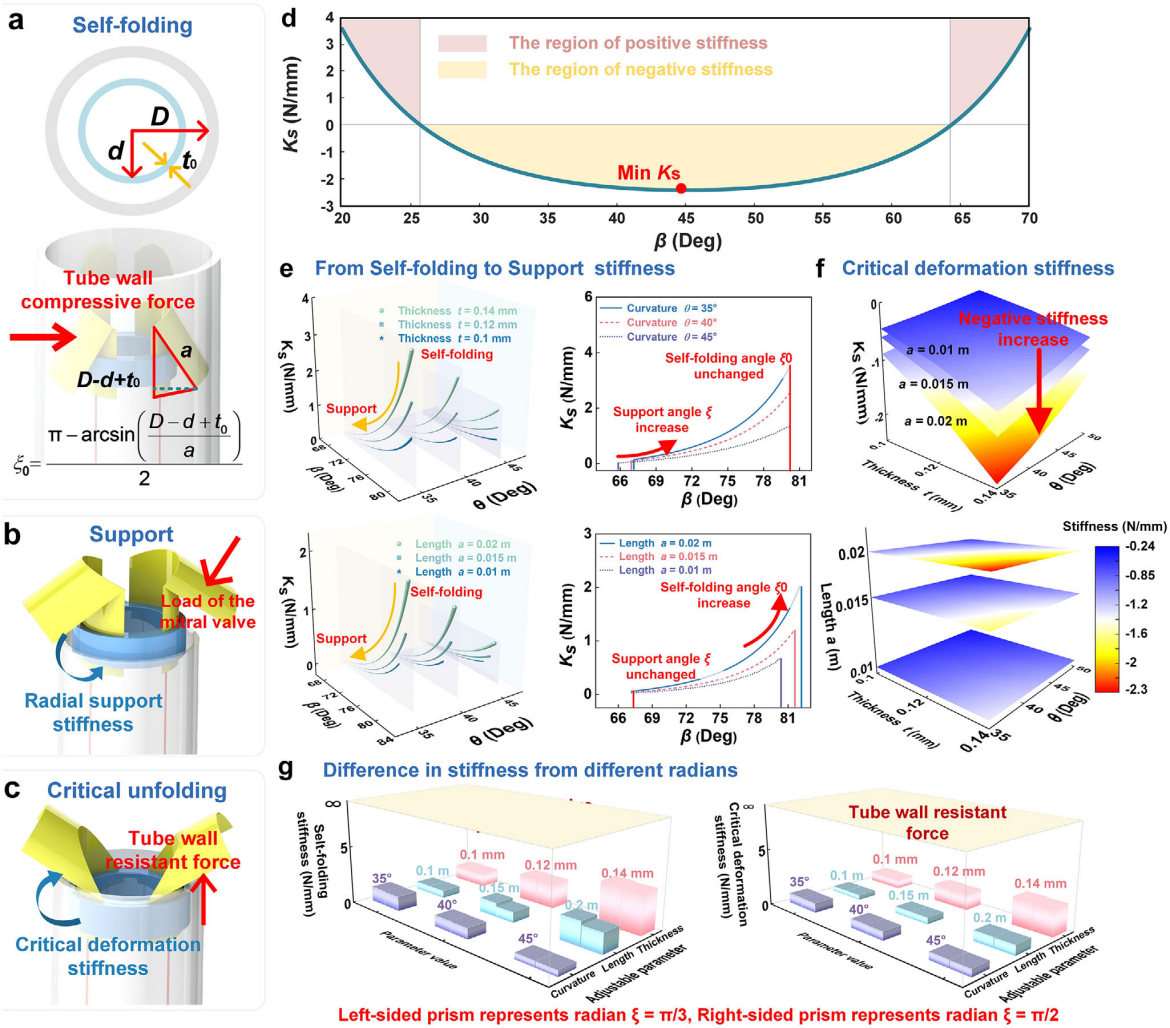
Design of radial stiffness of curved origami dilator: a–c)Three key points in the deformation of curved origami dilator; d) The full‐area equivalent stiffness of the curved origami dilator; e) Radial stiffness profile of the curved origami dilator transitioning from a self‐folding mode to supporting mode; f) Crtical deformation stiffness of the curved origami dilator of critical unfolding mode; g) Quantitative comparison of self‐folding stiffness and critical deformation stiffness differences between critical modes at ξ  =  π/3 and ξ  =  π/2, respectively.

**Figure** **4** investigates the influence of geometric parameters on the stiffness characteristics of the curved origami dilator across different deformation morphologies. The dilator exhibits two key transitional phases. The first occurs during the transformation from the self‐folding mode to the supporting mode, while the second takes place as the structure progresses from the supporting mode to the critical unfolding point before full deployment. The whole process is highly sensitive to variations in curvature, thickness, crease radius, and arc length of the curved origami dilator.

As shown in Figure 4a–c, each deformation morphology of the curved origami dilator corresponds to a distinct interaction mode between the structure, the tube wall, and the mitral valve. In the self‐folding mode (Figure 4a), the dilator is compressed by the surrounding tube wall, thereby storing elastic potential energy. The initial deformation angle of the curved surface can be described as:

(16) $$\xi =\frac{\pi -arcsin\left(\frac{D-d+t_{0}}{a}\right)}{2}\;$$

where *D*, *d*, and *t*_0_ represent the inner diameter of the tube, the inner diameter of the fixed hoop, and the wall thickness of the fixed hoop, respectively. During the transition from the self‐folding to the supporting mode (from Figure 4a,b), the dilator comes into contact with the mitral valve, providing radial support. When the valve contracts, the interaction force causes the curved origami dilator to alternate between the supporting mode (mechanically relaxed) and the self‐folding mode (fully stressed). Subsequently, when a clamp is used to grasp the valve, a pulling force is applied to the dilator, driving it into the critical unfolding mode (from Figure 4b,c). At this morphology, the structure is resisted by the tube wall, and its deformation behavior is characterized by negative stiffness, indicating a mechanically unstable transition regime essential for snap‐through deployment.

As shown in Figure 4d, within the yellow area, the system's equivalent stiffness is less than zero, resulting in local instability. At this point, a slight increase in the external displacement will cause a reverse force response. The system no longer absorbs energy but instead rapidly releases the stored elastic energy. Therefore, the area of negative stiffness can be regarded as an important design criterion for evaluating the safety and sensitivity of the curved origami dilator.

To further elucidate the deformation behavior during the transition from the self‐folding mode to the supporting mode, Figure 4e presents both 3D surface plot and a 2D projection of the radial support stiffness under varying geometric parameters. The results demonstrate that increasing either the arc length or the thickness leads to a significant enhancement in radial support stiffness, particularly when the spatial curvature angle θ remains constant. Conversely, increasing the curvature results in a reduction in stiffness, thereby reducing the stability of the supporting configuration.

In addition, the curvature alters the stable folding angle β of the supporting mode. A smaller curvature results in a larger supporting angle, meaning that the two folded surfaces move closer together. Furthermore, the initial angle of the dilator in the self‐folding mode is influenced by the origami length. Structures with higher curvature and greater length exhibit a broader tunable range of radial support stiffness, indicating stronger mechanical resistance and improved shape stability. These findings provide valuable design guidelines for optimizing the mechanical response of the curved origami dilator for biomedical applications.

Figure 4f further examines the critical deformation stiffness involved in the transition from the supporting mode to the recycling mode of the curved origami dilator. Within this transition region, distinct negative‐stiffness zones are observed, and these effects are particularly pronounced in configurations with shorter lengths and smaller thicknesses. As the curvature angle *θ* increases, the magnitude of the negative stiffness also increases. The stiffness at this critical unfolding point reflects the restoring force required by the tube wall to trigger an instantaneous geometric transition. A larger negative stiffness corresponds to a higher force threshold needed to drive the dilator back toward the retracted configuration. Finally, Figure 4g compares the self‐folding stiffness and critical deformation stiffness of the curved origami dilator with different crease radius configurations, specifically for ξ  =  π/3 (left prism) and ξ  =  π/2 (right prism). The analysis reveals that a smaller crease radius results in higher stiffness during both self‐folding and unfolding phases, particularly when the thickness and length of the structure increase. In contrast, the larger radius results in a softer stiffness, yielding a more compliant curved origami dilator. The yellow regions represent the compressive and resistive forces exerted by the tube wall. These restoring forces are fully mobilized to either constrain or facilitate the deformation of the dilator, depending on the relative stiffness of the structure in each morphological mode.

Overall, the stiffness behavior of the curved origami exhibits a high degree of tunability and design flexibility, enabling the structure to meet the dual requirements of mechanical stability and reversibility, which are essential for safe and effective operation in interventional medical applications.

From Figure 4, it can be observed that the curved origami dilator exhibits a distinct negative stiffness region. This region, spanning approximately (Δ*β*_crit_ ≈ 0.698 rad), corresponds to the snap‐through transition between the supported and recovery states. Within this range, the structure releases the stored elastic energy, resulting in a rapid geometric reconfiguration and force drop. To ensure mechanical safety during clinical operations, the relationship between the snap‐through threshold and the physiological thrust of the valve was quantified. Taking the maximum physiological load 6– 8 N and applying a safety factor is 2, the critical transition force was set to *F*_crit_ = 12–16 N. Using the equivalent force arm *L* = 8 mm, which is the folding length, and the measured angular transition range, the corresponding stiffness safety threshold was calculated as:

(17) $$K=F_{\mathrm{crit}}/\left(L\Delta \beta_{\mathrm{crit}}\right)=2.15-2.87\;N/\mathrm{mm}$$

It defines the mechanical safety range for the curved origami dilator, indicating that as long as the actual structural stiffness remains above this range, the device can provide sufficient support while avoiding premature snap‐through or excessive transient pressure on the valve tissue.

### 2.3 Experiments of the Curved Origami Dilator

**Figure 5:**
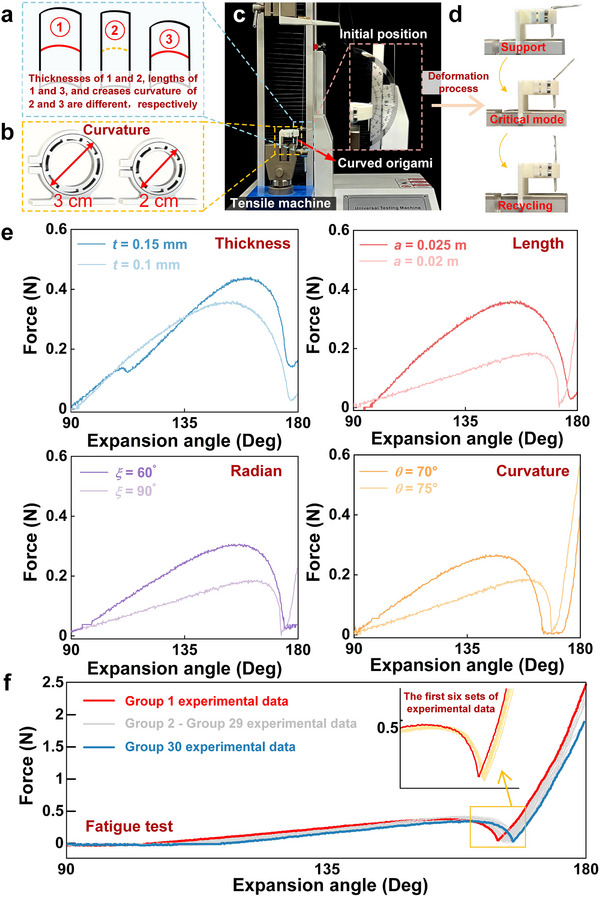
Experimental validation of the bistable behavior of the curved origami dilator: a) Curved origami with defined geometric parameters; b) Fixed hoop of different diameters; c) Experimental setup for the quasi‐static tension and compression test of the curved origami; d) Three representative deformation modes during loading: Self‐folding, support, and recycling; e) Restoring force under varying parameters; f) Curved origami fatigue test.

To verify the functionality of the curved origami dilator, **Figure** **5**a presents structures with different geometric parameters. As shown in Figure 5b, fixed hoops with diameters of 3 and 2 cm produce distinct initial curvatures. The test setup is shown in Figure 5c. Figure 5d depicts three typical modes during the loading process: the support mode, the critical unfolding mode, and the recycling mode. Initially, the curved origami dilator is folded with high deformation energy. As the load increases, it reaches the critical mode with significant geometric and nonlinear mechanical changes, then overcomes the energy barrier to achieve the second stable configuration.

Figure 5e systematically summarizes how different geometric parameters affect the mechanical properties of the curved origami dilator. The upper right corner of Figure 5e illustrates the influence of origami thickness on the force–displacement curves. It overcomes the potential energy barrier at the designed unfolding angle in the range of 140° − 150°, confirming the designed bistability. Thicker material (*t*  =  0.15 mm) shows a higher peak load and larger energy barrier, while the thinner one (*t*  =  0.1 mm) has a flatter response curve with less pronounced mechanical transitions, indicating lower structural stability. The upper left corner of Figure 5e discusses the influence of length on the mechanical behavior of the curved origami. The results show that as the length increases, the critical force required to drive unfolding rises, and the deformation range expands. This indicates that longer origami cells store more elastic energy, enhancing their ability to overcome the potential energy barrier and improving bistable stability. The bottom right corner of Figure 5e shows the effect of the crease radius. A smaller radian angle (α  =  60°) exhibits a greater jump displacement. The bottom left corner of Figure 5e illustrates the effect of initial curvature, and a smaller curvature (θ  =  70°) shows a steeper load response slope during the initial loading morphology and exhibits more pronounced jumping behavior. The effects of geometric parameters are crucial for optimizing the mechanical performance and bistability of the origami structure dilator, providing both theoretical insight and experimental validation for designing curved origami‐based TMVR expansion devices.

As shown in Figure 5f, the 30 overlaid force–displacement curves exhibit almost identical profiles, confirming excellent reproducibility of the bistable transition. The snap‐through force gradually decreased from 0.4236 N (1st cycle) to 0.3854 N (30th cycle), corresponding to a deviation of 9.1%, as summarized in **Table** **1**. No cracks, delamination, or plastic deformation were observed after testing, indicating stable mechanical integrity of the PET‐based origami structure. No visible cracks, delamination, or plastic deformation were observed after testing, demonstrating stable mechanical integrity of the PET‐based origami unit.

**Table 1:** Quantitative comparison of bistable performance over 30 cycles.

Cycle No.	Snap‐through force [N]	Deviation from 1st cycle [%]
1	0.4236	–
5	0.4103	3.1
10	0.3959	6.5
20	0.3863	8.8
30	0.3854	9.1

These results indicate that although the device is intended for single clinical deployment, its bistable mechanism maintains consistent mechanical performance under repeated actuation, ensuring reliability and safety within the single‐use operation.

The bistable snap‐through and self‐recovery behavior of the curved origami dilator is shown in Movie S1 (Supporting Information).

### 2.4 Experimental Device of the Curved Origami Dilator for TMVC

**Figure 6:**
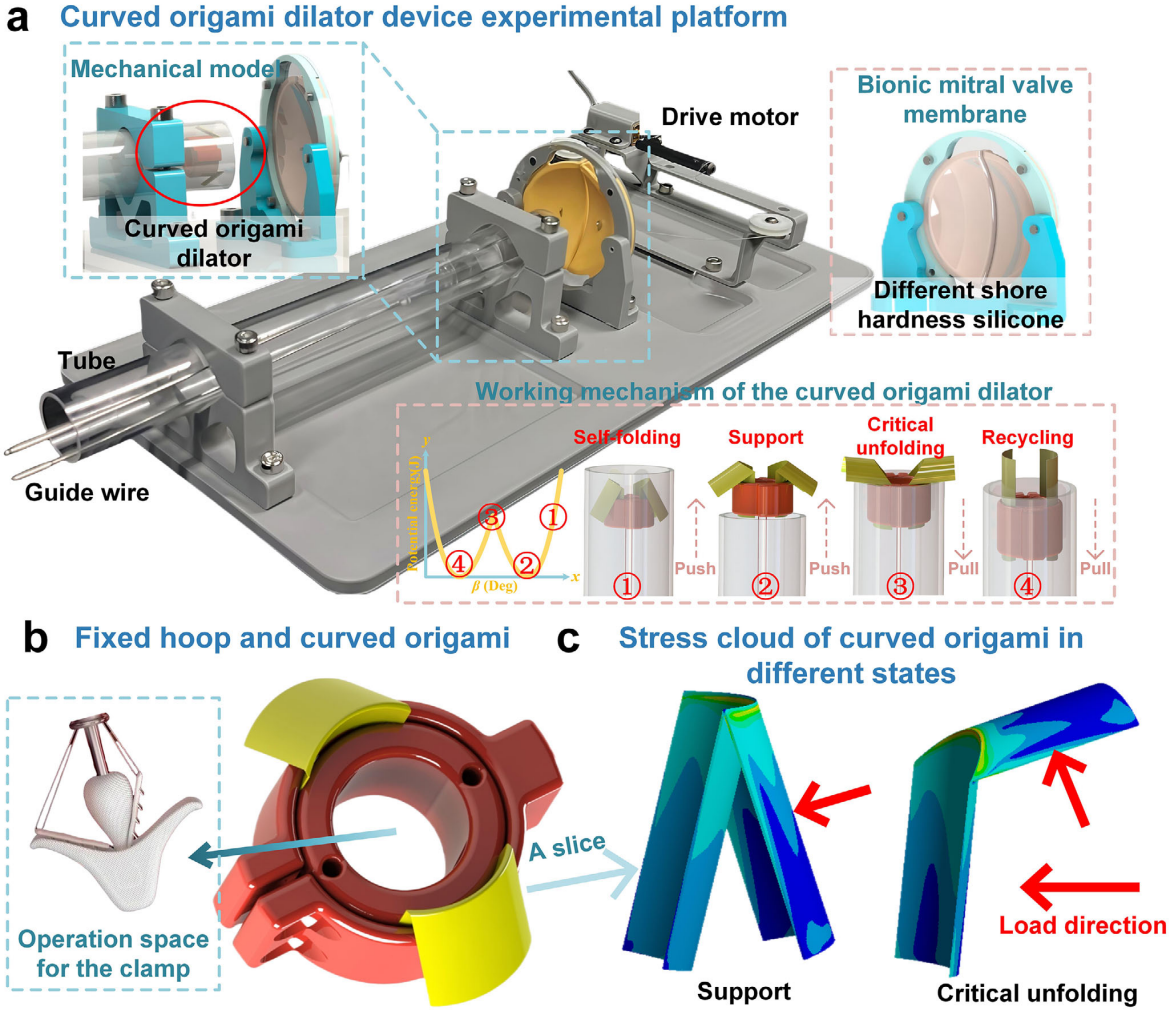
Mechanical model of the curved origami dilator for transcatheter mitral valve repair: a) Integrated test platform including the drive motor, bionic mitral valve membrane, and transparent tube; b) Structural design of the curved origami and fixed hoop; c) Finite element stress cloud plots.

In the previous section, numerical simulations and experimental results confirmed that the proposed curved origami dilator exhibits pronounced bistability, with its radial support stiffness tunable through key geometric parameters. Building on this theoretical design, a prototype expandable curved origami dilator for TMVC is fabricated (**Figure** **6**a). To evaluate its mechanical performance, unfolding tests are conducted under bionic mitral valves of varying stiffness, verifying the support stability and release reliability, and further demonstrating the practical viability of the theoretical design in clinically relevant scenarios.

As shown in Figure 6a, the curved origami dilator prototype for transcatheter mitral valve clamping comprises a drive motor, a bionic mitral valve, a curved origami dilator, and a transparent tube. The drive motor powers an axial push–pull motion that controls the opening and closing of the bionic mitral valve, which is fabricated from silicone with adjustable Shore stiffness and customizable geometry. The curved origami dilator, positioned on the left, is delivered via the catheter in a compact mode, enabling minimally invasive transport and self‐expansion at the target site. The lower part of the figure illustrates the bistable deformation path of the curved origami:

1. Initially, in a self‐folding compressed mode inside the tube.
2. Pushing the guidewire triggers self‐folding to the support mode, where the structure resists the bionic mitral valve contraction force through its radial stiffness, achieving dilation.
3. Further tension causes the curved origami dilator's backside to contact the tube wall. When the tube's resistance exceeds the origami's potential energy barrier, it reaches a critical unfolding mode.
4. Finally, it reverts to the recycling mode.

Figure 6b illustrates the spatial arrangement of the curved origami relative to the fixed hoop. The origami assembly not only provides effective support to the unfolded structure against the valve but also maintains sufficient clamping space, ensuring unobstructed access for subsequent valve anchoring. This design guarantees reliable deployment and repositioning during the surgical procedure.

Additionally, to investigate the stress behavior of the bistable curved origami under different deformation modes, Figure 6c presents the stress distribution maps in both the self‐folding and critical unfolding modes. Finite element simulations were performed in Ansys. The PET film (*t* = 0.1 mm) was modeled as a linear elastic material with *E* = 4 GPa. The 3D geometry was meshed using shell elements with local refinement near the creases and a coarser mesh in the surrounding area. The simulation fully describes a situation where one side has a fixed boundary (fully constraining the nodes at the circular boundary), while the other side is a free boundary. The loading is applied according to displacement control to replicate the loading path as observed in the experiment. The results indicate that initial stresses are primarily concentrated around the crease regions. As the curved origami unfolds further, the internal stresses gradually propagate along the crease lines, forming distinct high‐stress bands. This observation validates the designed deformation path of the structure.

After completing the design and fabrication of the curved origami dilator prototype for TMVC, further in situ performance tests were conducted. By simulating key procedural steps—such as transcatheter delivery, support, and recycling—the mechanical stability of the bistable curved origami dilator within the clinical workflow was validated.

**Figure 7:**
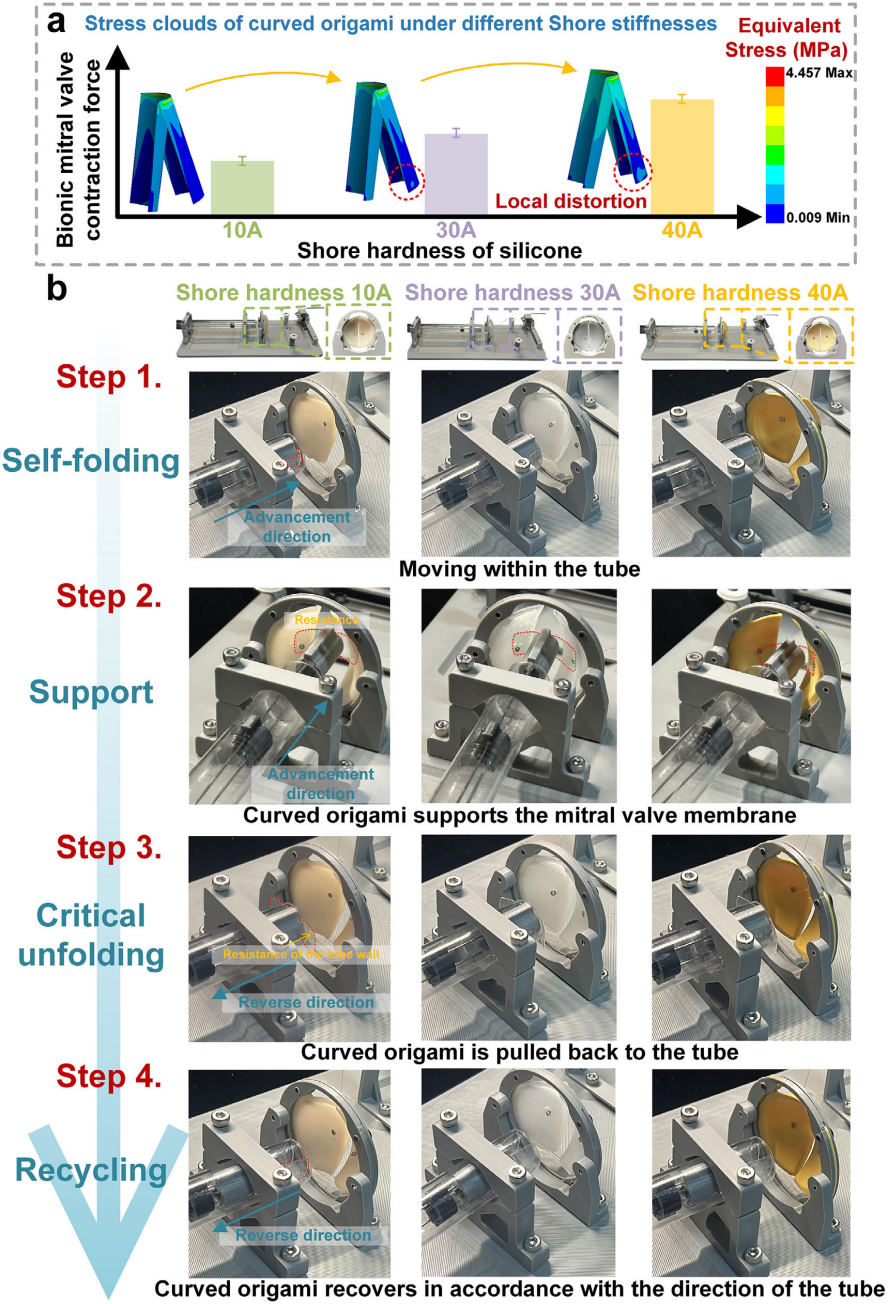
Stress clouds and functional demonstration of curved origami dilator under different silicone Shore hardness conditions: a) Equivalent stress distribution of curved origami; b) Functional demonstration of curved origami dilator.

As shown in **Figure** **7**a, we compared the contraction forces exerted by the bionic mitral valve during the unfolding process of the curved origami dilator and performed finite‐element simulations to generate corresponding stress‐distribution maps. As the silicone's Shore hardness increased from 10 to 40 A, the contraction force exerted by the structure progressively increased. However, when the hardness reached 40 A, stress concentration and localized deformation were observed in the curved origami, indicating that excessive stiffness of the mitral valve may reduce the deformation coordination capacity of the curved origami dilator. Therefore, in practical applications, the selection of the curved origami should be tailored to the severity of the mitral valve lesion, balancing the need for sufficient structural stiffness with deformation adaptability.

Figure 7b illustrates the four key steps of the curved origami dilator's expansion process for bionic mitral valves with varying Shore hardnesses, along with a comparative analysis. In Step 1, the curved origami dilator is in a self‐folding mode, smoothly advancing through the catheter. In Step 2, the drive motor applies axial thrust to control the opening and closing motion of the bionic mitral valve, simulating the function of a real mitral valve. This drives the curved origami dilator to unfold and reach the target position within the valve. The dilator effectively props open the valve with sufficient radial support, especially in valves with a Shore hardness of 40A, demonstrating enhanced unfolding capability. In Step 3, the dilator completes the propping process and reaches a critical instability upon contacting the tube wall. Finally, in Step 4 (recycling mode), the curved origami dilator returns to the recycling mode as reverse tension is applied by the drive system, showcasing its reversible deformation and recovery capability. The process was successfully completed under three different Shore stiffness conditions, demonstrating the structure's excellent stability and repeatability across multiple cycles.

The functional assessment of transcatheter mitral valve dilation is demonstrated in Movies S2–S4 (Supporting Information), using a biomimetic heart model with valve hardness values of 10, 30, and 40 A, respectively. These videos show the delivery, deployment, and dilation sequences under different valve stiffness conditions.

### 2.5 In Vitro Experiment on the Curved Origami Dilator for TMVC

**Figure 8:**
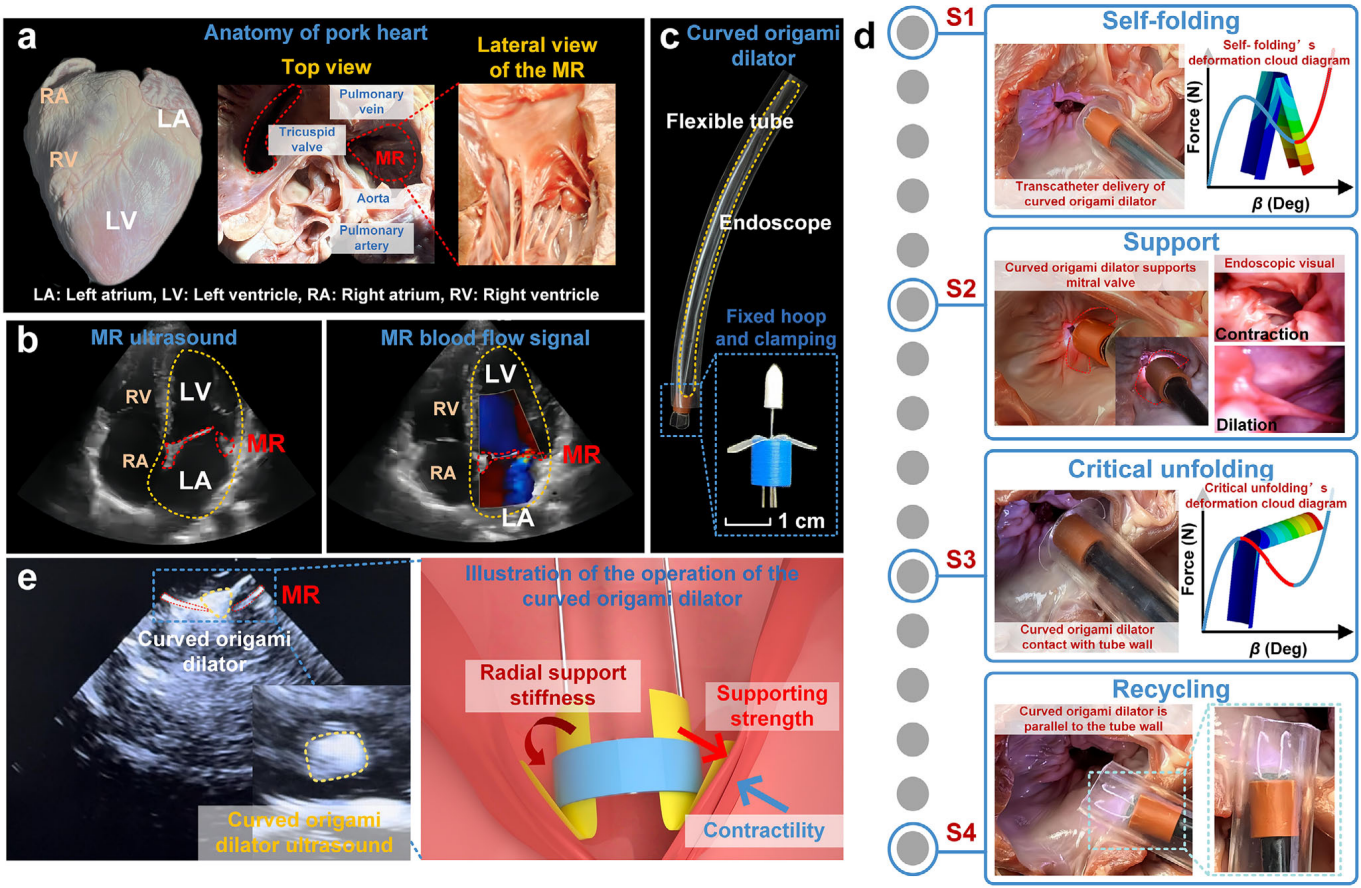
Ex vivo demonstration of the transcatheter deployment and retrieval process of the bistable curve origami structure in a pig heart: a) Pig heart and mitral valve; b) Echocardiography of mitral valve disease; c) Curved origami dilator; d) Four‐step operations; e) The ultrasonic and mechanical schematic diagram of the curved origami dilator.

After completing the functional validation of the curved origami dilator in the simulated transcatheter mitral valve, further ex vivo experiments were conducted using a porcine heart model to evaluate the device's practical operability and mechanical performance in a biologically relevant environment, as shown in **Figure** **8**.

As shown in Figure 8a, the position of the mitral valve of the pig's heart and the location of the dilator are determined. The top and side views clearly depict the left atrium (LA), left ventricle (LV), right atrium (RA), and right ventricle (RV), and mark the mitral valve (MR) area as the target area for intervention. Figure 8b shows ultrasound imaging and Doppler blood flow signals, confirming the presence of regurgitation between the left atrium and left ventricle, providing a physiological background for evaluating the support function of the dilator. Figure 8c shows that the curved origami dilator assembly consists of a flexible catheter, an endoscope, a fixed hoop, and the curved origami structures. During the procedure, the system advances the curved origami dilator through the left atrial pathway to the mitral valve target area. For precise experimental control, an endoscope is integrated within the catheter to provide real‐time visual guidance.

To visualize the working modes of the curved origami dilator in an isolated porcine heart across different deformation morphologies, the experimental procedure was divided into four key steps, corresponding to the entire process of self‐folding, support, critical unfolding, and recycling of the dilator, as illustrated in Figure 8d.

First, the curved origami dilator starts in an initial self‐folding mode and is slowly advanced through the flexible tube to the mitral valve position in the porcine heart. In this mode, the dilator's overall volume is small and compact, ensuring smooth transcatheter delivery. The curved origami remains in a low‐potential‐energy mode, providing excellent system stability and spatial adaptability.

Next, continued thrust pushes the curved origami dilator out of the tube, releasing the constraints imposed by the tube walls. This action releases the internal stresses within the origami, prompting a spontaneous transition from the self‐folding mode to the support mode. This unfolding behavior is driven by the bistable geometry of the design rather than an external force. Once the curved origami dilator reaches the target mitral‐valve position and stops advancing, it exerts a significant radial support force on the valve tissue. The mitral valve is observed to be propped open, effectively mimicking the expansion of a stenotic orifice during surgery. Endoscopic images captured during this morphology verify the biocompatibility and reliable support performance of the structure within the stereotaxic porcine heart.

Upon completion of expansion, the fixed hoop is actuated to retract the curved origami dilator, initiating the recovery phase. Due to the radial resistance from the tube wall, the backside of the curved origami dilator experiences a constraining force, causing geometric refolding as it transitions from the supported mode back to the recycling mode. The recovery force curve shown in Step 3 illustrates the energy release during this steady‐mode transition.

Finally, the dilator fully retracts into the flexible tube, returning to cycling mode. This completes the closed‐loop expansion cycle of the curved origami dilator. The catheter and dilator are smoothly withdrawn from the body without causing tissue interference or leaving any residual structures. Step 4 illustrates the curved origami dilator after it has cleared the mitral‐valve region and been fully retracted into the catheter, confirming its excellent reversible bistability and recovery capability.

Figure 8e further confirms the successful deployment of the dilator via ultrasound imaging. The presence of the device at the mitral valve region is clearly visualized, and its structural integrity is maintained throughout the actuation. The schematic on the right illustrates the interactions. The dilator creates a radial support stiffness that resists the contractile motion of the mitral valve and facilitates unobstructed, contact‐free clamping of the valve after passing through the fixed hoop.

### 2.6 Potential Application

**Figure 9:**
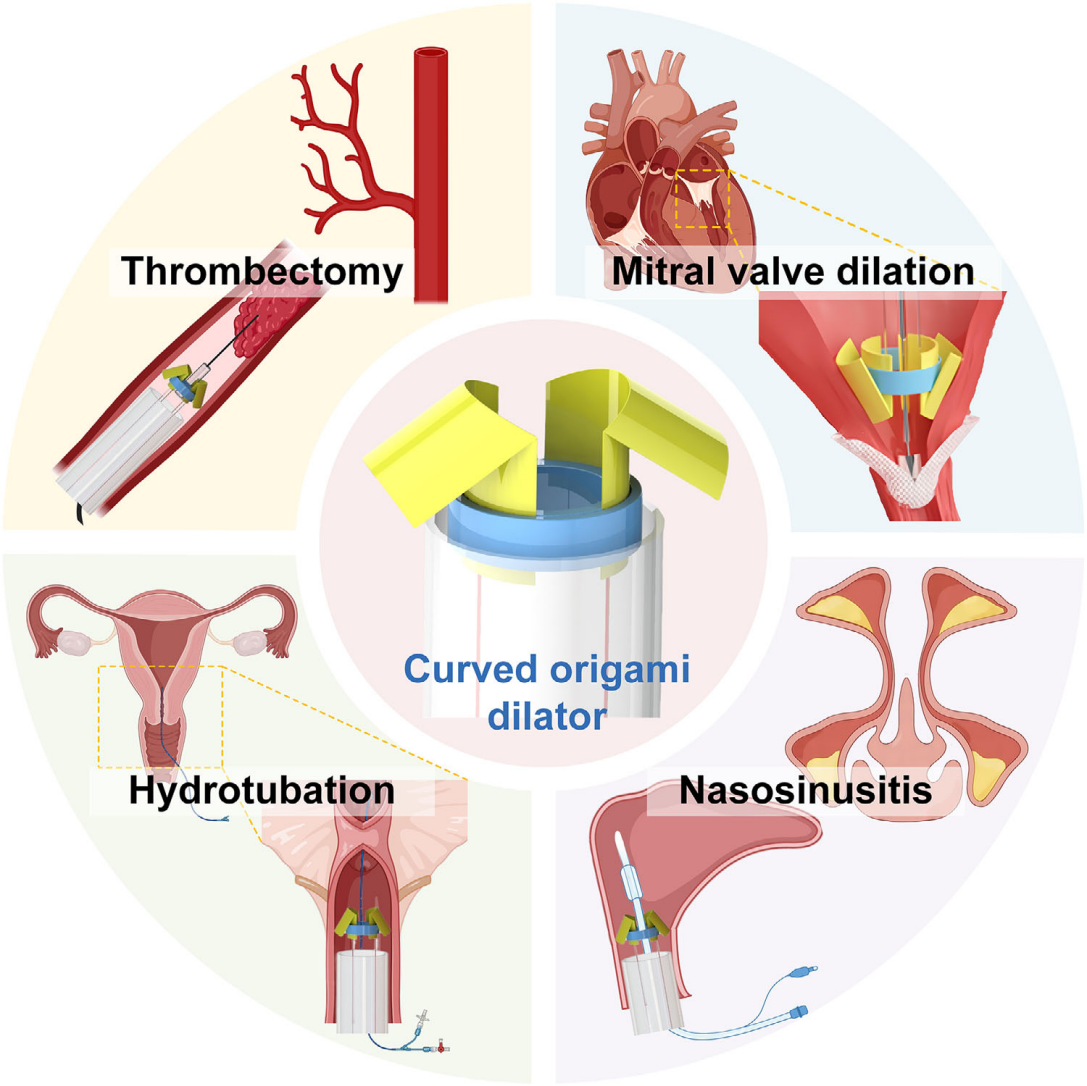
Multiple minimally invasive interventional applications of the curved origami dilator.

Furthermore, the curved origami dilator has many more applications in minimally invasive interventional surgeries. Leveraging its compact, compliant, and reversible deformation characteristics, this design offers substantial versatility for a wide range of clinical scenarios beyond transcatheter mitral valve clamping. As illustrated in **Figure** **9**, the dilator can be readily adapted for procedures such as thrombectomy in narrow vessels, endoscopic dilation of the nasal sinuses in nasosinusitis treatment, and fallopian tube flushing (hydrotubation) in gynecological interventions. In these applications, the dilator's mechanical adaptability and controllable deployment ensure atraumatic contact with soft tissues, while its bistable behavior enables precise and repeatable expansion or retrieval. These features highlight its strong potential as a platform technology for next‐generation soft robotic instruments in the broader field of interventional medicine.

## 3 Conclusion and Discussion

To mitigate the risk of valve damage arising from imprecise positioning and release of the holder during a single transcatheter mitral valve clamping operation, we designed and validated a bistable curved origami dilator that meets the requirements for lightweight construction, reversibility, and high stability, making it well‐suited for TMVC applications. Based on the principle of minimum potential energy, we developed a mechanical theoretical model of the curved origami, unveiling its folding–unfolding bistable transition mechanism and deriving analytical expressions for the origami's total potential energy and equivalent stiffness. Numerical analysis indicated that geometric parameters strongly influence the potential energy barrier height and bistability performance, thereby determining the support stability and energy‐storage capacity of the origami structure. Static tensile tests show an agreement trend with theoretical predictions, confirming that the curved origami dilator undergoes a jump‐like deformation from folded to unfolded modes, while maintaining outstanding structural integrity and morphological recovery. Moderate increases in the thickness and length of the origami enhance the potential energy barrier height and the amplitude of negative stiffness, thus improving bistability performance. Meanwhile, smaller initial curvature and crease radius contribute to increased radial support stiffness and enhanced mechanical responsiveness. Furthermore, we established an ex vivo experimental platform to demonstrate the functionality and biocompatibility of the curved origami dilator. The results demonstrated that the structure exhibits high design flexibility, stable response behavior, and excellent reversibility throughout deformation, underscoring its potential for application in the mechanical design of expandable medical devices and providing a robust theoretical foundation supported by experimental validation.

Compared with the existing TMVR and TEER products that use rigid metal frames and high clamping force to fix the valve leaflets, the proposed curved origami transcatheter mitral valve clamping introduces a soft bistable origami structure that can gently support the mitral valve rather than tightly clamping the valve tissue. This novel valve support method has several key advantages: a smaller delivery size (26–28 Fr), facilitating vascular access; a reversible unfolding method that allows precise control of the ejection transition; and single‐use, which minimizes local stress and potential tissue damage. By stabilizing the valve annulus without permanent fixation, the curved origami expansion device provides an open working space for subsequent repair or replacement surgeries, enhancing surgical flexibility and reducing overall surgical risk. Overall, these characteristics indicate that the curved origami TMVC performs well in terms of mechanical safety and operational adaptability, and is a promising next‐generation solution for minimally invasive mitral valve interventional therapy.

## 4 Experimental Section

### The Design and Material Selection of Curved Origami

The curved origami dilator is a single‐use expandable device made of medical‐grade PET, ensuring mechanical stability and short‐term biocompatibility during clinical operation. Cyclic testing showed less than 10% variation in force–displacement curve and no structural damage, confirming reliable bistable performance within its intended use. Although long‐term fatigue was not required, future optimization of crease geometry and material selection—such as adopting larger crease radian, or TPU/Parylene‐coated PET—may further enhance durability and safety for potential extended applications.

### Tensile Test of Bistable Curved Origami

The curved origami (Figure 5a) and the fixed hoop (Figure 5b) with precise geometry were fabricated through a hybrid process combining CAD design, photolithography, and 3D printing. The geometric model of the bending origami unit was created using CAD software. The folding curvature, curvature radius, and unit size were defined parametrically to achieve the desired bistable structure. The designed pattern was transferred to a medical‐grade PET film (t = 0.1–0.15 mm) through the lithography process, while the fixed hoop was independently manufactured using a 3D printer (XY resolution = 25 µm; layer thickness = 150 mm). After printing, it was assembled into a complete bending origami dilator, providing a framework for the bistable deformation.

The testing platform (Figure 5c) was a single‐column electronic tensile machine with stable loading and high‐sensitivity data acquisition. The clamp's lower end was rigidly mounted to the tester's lower fixture to ensure uniform loading. The bistable origami cell was initialized with a folding angle α  =  90°, and subjected to an upward axial load to evaluate its deformation behavior under quasi‐static conditions. The upper fixture moved downward at a constant speed of 10 mm min^−1^, while force–displacement curves were recorded in real time by a high‐precision sensor. This setup minimizes dynamic inertia interference, ensuring accurate and reliable data for subsequent bistable response analysis.

### Preparation of Bionic Mitral Valves

To replicate the mechanical environment of mitral valves under physiological conditions, bionic valve components were fabricated using a casting method with medical‐grade silicone of varying Shore hardness, selected for their excellent biocompatibility and elastic recovery. To assess the support performance of the curved origami structure across valves of different stiffness, all bionic valves were made with identical shape and dimensions (diameter and thickness), varying only in silicone hardness. In the fabrication process, inverted molds were 3D printed, and well‐mixed silicone was poured into the mold cavity, vacuum‐defoamed to ensure uniformity, and cured at room temperature or in a low‐temperature oven. The result was a series of structurally stable, highly reproducible mitral valve models with tunable mechanical properties.

### Functional Demonstration of a Bistable Curved Origami Expander for TMVC

The experimental system consisted of three main components: a drive motor (right), a bionic mitral valve (center), and a transcatheter curved origami dilator (left). The drive motor provides a stable axial force, driving the simulated mitral valve to cyclically open and close via a transmission mechanism, thereby replicating physiological heart valve motion. The dilator, based on the bistable curved origami developed in this study, was delivered via a catheter (inner diameter 12 mm, wall thickness 1 mm).

### In Vitro Experiment

The experiment used pig heart specimens. The expansion system consisted of a flexible catheter (inner diameter 8mm, wall thickness 1mm), an endoscope, a fixed hoop, and pre‐installed curved origami.

## Conflict of Interest

The authors declare no conflict of interest.

## Supporting information



Supplemental Movie 1

Supplemental Movie 2

Supplemental Movie 3

Supplemental Movie 4

Supporting Information

## Data Availability

The data that support the findings of this study are available from the corresponding author upon reasonable request.
