# Supplementary material for: Curved Bistable Origami‐Inspired Flexible Transcatheter Mitral Valve Clamping
Source: Adv Sci (Weinh). 2025 Nov 25;13(8):e17350. doi: 10.1002/advs.202517350 (PMC12884735; doi:10.1002/advs.202517350)
Supplement: Supplementary file 5 — Supporting Information [file ADVS-13-e17350-s003.docx]

ADVANCED SCIENCE


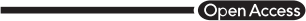


Supporting Information

Curved Bistable Origami-Inspired flexible Transcatheter mitral valve clamping

*Siyu Gao, Fan Jiang, Xiuting Sun*,Peng Qi, Jian Xu*

**Supporting Information**

**Curved Bistable Origami-Inspired flexible Transcatheter mitral valve clamping**

Siyu Gao, Fan Jiang, Xiuting Sun*,Peng Qi, Jian Xu

*Email of Corresponding Author: [05mech_sunxiuting@tongji.edu.cn](mailto:05mech_sunxiuting@tongji.edu.cn)

**Supplementary Figure**

**Figures 1-9 and Toc Figure**

**Supplementary Movie Captions**

**Movie S1. Verification of the bistable performance of surface origami.** The tensile test verified that the curved surface origami has a distinct bistable property and a negative stiffness region.

**Movie S2. Functional demonstration for transcatheter mitral valve dilation surgery for a biomimetic model with 40A valve hardness.**  Functional assessment of transcatheter mitral valve dilationon a biomimetic model with 40A valve hardness.The curved surface origami dilator can achieve stable release, reliable support and safe recovery.

**Movie S3. Functional demonstration for transcatheter mitral valve dilation surgery for a biomimetic model with 10A valve hardness.** The curved surface origami dilator can achieve stable release, reliable support and safe recovery.

**Movie S4. Functional demonstration for transcatheter mitral valve dilation surgery for a biomimetic model with 30A valve hardness.** The curved surface origami dilator can achieve stable release, reliable support and safe recovery.
